# Reversible Orbital Apex Syndrome

**DOI:** 10.3390/jemr19030043

**Published:** 2026-04-27

**Authors:** Yakov Rabinovich, Inbal Man Peles, Zina Almer, Iris Ben Bassat-Mizrachi, Jonathan Sapir, Noa Hadar, Alon Zahavi, Nitza Goldenberg-Cohen

**Affiliations:** 1 Department of Ophthalmology, Bnai Zion Medical Center, Haifa 3339419, Israel; rayakov@gmail.com (Y.R.); pelesman@gmail.com (I.M.P.); noahadar78@gmail.com (N.H.); 2 Department of Ophthalmology, Shamir Medical Center, Zerifin 7030001, Israel; almerzina@gmail.com; 3 Faculty of Medical and Health Sciences, Tel Aviv University, Tel Aviv 6997801, Israel; irisbenbassatmizrachi@gmail.com (I.B.B.-M.); alonzahavi@gmail.com (A.Z.); 4 Department of Ophthalmology, Sheba Medical Center, Tel Hashomer, Ramat Gan 5262000, Israel; 5 Department of Radiology, Bnai Zion Medical Center, Haifa 3339419, Israel; jonathan.sapir@b-zion.org.il; 6 Department of Ophthalmology, Laboratory of Eye Research, Felsenstein Medical Research Center, Rabin Medical Center, Petach Tikva 4941492, Israel; 7 The Krieger Eye Research Laboratory, Faculty of Medicine, Technion—Institute of Technology, Haifa 3200003, Israel

**Keywords:** apex syndrome, reversible ophthalmoplegia, orbital apex syndrome, optic neuropathy, extraocular motility disorder

## Abstract

Orbital apex syndrome (OAS) is characterized by optic neuropathy and ophthalmoplegia and is generally associated with poor visual prognosis. The aim of this study was to describe patients with acute OAS who demonstrated substantial recovery of visual function and ocular motility. We retrospectively reviewed the medical records of patients treated for OAS at a tertiary medical center between 2019 and 2024 whose condition ultimately proved reversible. Data on demographics, clinical findings, imaging, management, and follow-up were collected. Six patients (three female, three male; age range 14–87 years) were included and followed for a median follow-up of 7 months (range 2–31). All presented with reduced vision and ophthalmoplegia of varying severity. Underlying etiologies included inflammatory disease (n = 2), lymphoma, infection, blunt trauma, and post-surgical OAS of undetermined etiology (n = 1 each). Treatment was directed at the underlying cause. Visual acuity ranged from 20/30 to hand motion (HM) at presentation and 20/15 to 20/60 at the final visit. Improvement in vision and ocular motility occurred after a median time to clinical improvement of 2.37 months (range 0.25–5 months). Near-complete recovery of ocular motility was observed in all patients, with only one retaining mild abduction limitation. These findings highlight a subset of OAS cases with favorable outcomes and emphasize the importance of early diagnosis and etiology-directed management.

## 1. Introduction

The orbital apex is a narrow anatomical space that contains vital neural and vascular structures. The convergence of critical structures within such a confined space makes the orbital apex susceptible to compressive pathologies [1,2]. Orbital apex syndrome (OAS) is a severe condition characterized by a combination of optic neuropathy and ophthalmoplegia involving several cranial nerves (CNs), including the optic nerve (CN II), motor cranial nerves (CNs III, IV, and VI), and nasociliary branch of the ophthalmic division (V1) of the trigeminal nerve (CN V) [2]. The etiologies of OAS are diverse, including infectious, inflammatory, neoplastic, and vascular pathologies, and direct or blunt trauma [2,3,4,5,6]. According to several case series, neoplasms account for approximately half the cases [4,5]. The reported visual prognosis is usually poor, with a final outcome worse than 20/200 in 57% to 77% of patients; only one-third of patients regain good visual acuity (VA) [4,5].

Tumor invasion of the narrow bony passageway of the apex affects all nerves that pass through it to the orbit [2]. The major effect is a decline in VA, in 80–86% of patients, followed by proptosis and ophthalmoplegia [4,5]. All three symptoms are reported in 54% of patients [4]. Additionally, patients may experience ptosis and sensory alterations in the forehead, eyelid, and upper nose due to involvement of CN VI [4]. An acute presentation of impaired vision and of ocular motility, particularly when rapidly progressive, should raise suspicion of OAS. Prompt clinical evaluation, imaging, and intervention are essential to prevent permanent damage.

Data on the duration of OAS symptoms and time to clinical improvement of extraocular motility remain scarce. One long-term follow-up study of OAS due to nasopharyngeal rhabdomyosarcoma reported an average time to clinical improvement of 2.2 months with high variance [7]. However, final VA was poor, with two-thirds of patients failing to improve [7]. In another study of OAS of diverse etiologies, the average duration was 1.25 months [4].

Since OAS is a common final pathway of different etiologies, accurate diagnosis and effective treatment rely heavily on imaging studies. Computerized tomography (CT) is crucial for identifying bone abnormalities, acute hemorrhages, and masses. Magnetic resonance imaging (MRI) is often required for detailed characterization of the lesion, including its extent and the involvement of surrounding soft tissues [6]. Biopsies from this region are highly difficult to obtain and dangerous.

Treatment of OAS is aimed at the underlying cause. It often necessitates a multidisciplinary approach, and in infectious etiologies, it may involve a broad antimicrobial coverage [2].

Although good clinical outcomes are expected in some cases, this subset of patients is poorly characterized in the literature. The aim of the present case series was to describe a selected subset of patients with reversible OAS across diverse etiologies, in order to better characterize their clinical course, recovery patterns, and outcomes. While OAS is generally associated with poor prognosis, this subset of patients remains underrepresented in the literature. By focusing specifically on reversible cases, this study aims to describe the clinical course, recovery patterns, and outcomes observed in this subset and to highlight the importance of early recognition and etiology-directed management.

## 2. Materials and Methods

### 2.1. Study Design and Case Identification

This retrospective case series was conducted at a tertiary medical center and included patients evaluated between January 2019 and December 2024. Cases were identified through retrospective review of patients who presented to the neuro-ophthalmology clinic during the study period. The medical records and imaging studies of these patients were then reviewed individually to confirm fulfillment of the predefined clinical and radiologic criteria for orbital apex syndrome and documented clinical improvement during follow-up. No systematic search of hospital-wide databases, imaging registries, or diagnostic coding systems was performed. This study was not designed as a consecutive case series; rather, it represents a selected cohort of patients with reversible presentations of OAS. A complete denominator of all OAS cases evaluated during the study period was not systematically captured.

### 2.2. Inclusion and Exclusion Criteria

Patients were included if they presented with acute findings consistent with OAS, defined as optic neuropathy (reduced visual acuity and/or relative afferent pupillary defect), together with ophthalmoplegia involving one or more cranial nerves (III, IV, or VI), with or without involvement of the ophthalmic branch of the trigeminal nerve (V1). Additional inclusion criteria required radiologic evidence of pathology involving the orbital apex or adjacent structures on computed tomography and/or magnetic resonance imaging, and documented improvement in visual acuity and ocular motility during follow-up, with complete or near-complete ocular motility recovery at the final examination. Patients with incomplete medical records, insufficient follow-up, or absence of documented clinical improvement were excluded. A minimum follow-up duration of 1 month was required to ensure adequate assessment of clinical recovery.

### 2.3. Definition of Reversible Orbital Apex Syndrome

Reversible OAS was defined as documented improvement in visual acuity from presentation to follow-up together with complete recovery or near-complete recovery of ocular motility on follow-up examination. Near-complete recovery was defined descriptively as residual mild limitation in one direction of gaze or diplopia limited to specific gaze.

### 2.4. Diagnostic Evaluation

All patients underwent comprehensive ophthalmologic and neurologic evaluation, including assessment of visual acuity, pupillary responses, ocular motility, and anterior and posterior segment examination. Neuroimaging with computed tomography and/or magnetic resonance imaging was performed in all cases to assess involvement of the orbital apex and adjacent structures. Additional diagnostic workup, including laboratory testing, infectious evaluation, and systemic assessment, was performed according to the clinical context. Histopathologic confirmation was obtained when feasible and clinically indicated.

Etiologic classification in each case was determined based on integration of clinical presentation, imaging findings, laboratory and systemic evaluation, and, when available, histopathologic confirmation. In cases where tissue diagnosis was not feasible, the presumed etiology was based on the overall clinical course, including response to treatment and exclusion of alternative causes.

No standardized scoring system was used to quantify the severity of ophthalmoplegia or optic neuropathy; assessments were based on detailed clinical examination, including documentation of ocular motility deficits and visual function.

### 2.5. Data Collection and Follow-Up

Data were extracted from medical records and included demographic characteristics, presenting symptoms, cranial nerve involvement, imaging findings, presumed etiology, treatment modalities, time to clinical improvement, and duration of follow-up. Surgical treatment in this study refers to supportive or emergent procedures performed as part of acute management (e.g., canthotomy or sinus drainage), rather than definitive ocular motility or strabismus surgery. Patients were followed longitudinally with serial clinical examinations, and final outcomes were determined based on visual acuity and ocular motility at the last documented visit. Clinical improvement was determined based on comparison with baseline findings, including visual acuity and extent of ocular motility limitation.

### 2.6. Statistical Analysis

Given the descriptive design of the study, statistical analysis was limited to descriptive measures. Continuous variables are presented as medians and ranges, and categorical variables are summarized as counts and percentages. Formal comparative or correlation analyses were not performed due to the limited sample size and heterogeneity of etiologies, as such analyses would be underpowered and potentially misleading. No *p*-values or confidence intervals are reported.

## 3. Results

Three female and three male patients aged 14 to 87 years (median 65.5 years) were diagnosed with acute OAS. Their demographic and clinical characteristics are shown in Table 1. OAS etiologies included inflammation in two cases, and lymphoma, infection, blunt trauma, and post-surgical OAS of undetermined etiology in one patient each. The clinical diagnosis in all cases was based on findings of reduced vision and ophthalmoplegia at presentation. Three patients also had proptosis. One patient had documented reduced sensation in the V1 region. The median duration of follow-up was 7 months. During this period, the mother of patient 1 presented with a similar event that was suspected to be OAS.

Gradual improvement was noted over time, with the sequential order of nerve recovery documented for each patient, as shown in Table 2. At the end of follow-up, VA had improved in all cases, with near-complete recovery of extraocular motility, except for a residual limitation in abduction in one patient. VA improved from 20/30–HM at presentation to 20/15–20/60 at the end of follow-up. One patient had a known amblyopic eye.

### 3.1. Case Presentations

#### 3.1.1. Case 1: Recurrent Familial Episodes of Ophthalmoplegia

A 25-year-old male experienced recurrent episodes of alternating CN VI palsy following common colds, initially resolving spontaneously. Two years after the initial episodes, he presented with left ophthalmoplegia, ptosis, and proptosis without pupillary involvement, accompanied by slight visual decline (20/30). Family history revealed that his 61-year-old mother had experienced a similar episode four years earlier, initially described as “transient diplopia”, but documented as complete ophthalmoplegia that resolved spontaneously within two months; this observation is anecdotal, as she was not formally included in the study cohort.

**Figure 1:**
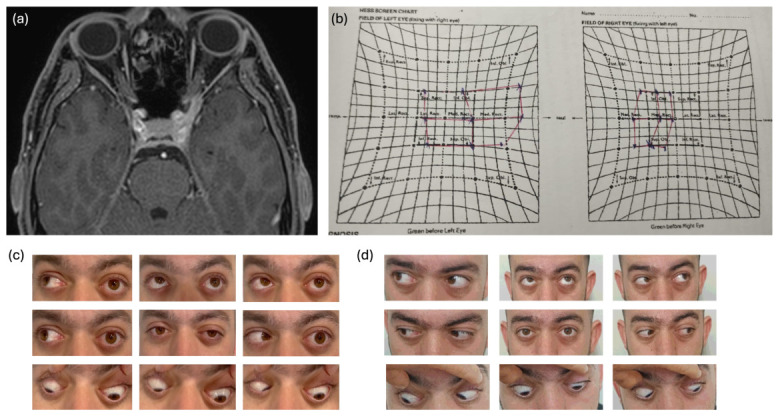
Case 1. (**a**) Contrast-enhanced, fat-suppressed T1-weighted MRI showing infiltrative involvement of the left orbital apex with proptosis. (**b**,**c**) Hess screen and extraocular motility testing at presentation demonstrating near-complete left ophthalmoplegia. (**d**) Follow-up motility examination showing marked recovery of extraocular movements, consistent with reversible orbital apex syndrome.

Comprehensive workup was mostly normal, except for elevated immunoglobulin E (IgE), peripheral eosinophilia, and herpes simplex virus 1 (HSV1) seropositivity. MRI showed an infiltrative lesion at the left cavernous sinus and orbital apex (Figure 1a). He improved significantly following steroid treatment (Figure 1b–d). The presumed inflammatory etiology was based on imaging findings, laboratory abnormalities (eosinophilia and elevated IgE), and the marked clinical response to corticosteroid therapy, in the absence of evidence for infectious or neoplastic disease.

A few months after her son’s recent episode, another episode of ophthalmoplegia was documented with his mother, that was spontaneously resolved. Initial workup included a CT scan without contrast, which was interpreted as normal.

Her medical history was positive for well-controlled diabetes and hypertension, as well as a hiatal hernia managed with omeprazole. On diagnostic workup, blood tests were negative for acetylcholine receptor antibodies, thyroid function was normal, and Hemoglobin A1c was 7.8% with elevated eosinophils, comparable to the son. Two months later, the mother reported complete absence of diplopia and full recovery of ocular movements, as evidenced by the documentation of normal optic nerve function.

#### 3.1.2. Case 2: Inflammatory OAS, Suspected Tolosa–Hunt Syndrome

An 87-year-old man presented with progressive left eye visual decline, ophthalmoplegia, ptosis, and proptosis together with reduced VA to hand motion (HM). Extensive neuroimaging (MRI, CT, CT angiography) revealed an infiltrative process involving the left cavernous sinus, orbit, and adjacent sinuses. Blood tests showed elevated C-reactive protein with otherwise unremarkable systemic workup. Infectious etiologies were excluded. Treatment with oral prednisone led to marked visual improvement from HM to 20/40. The ophthalmoplegia resolved. Follow-up MRI showed reduced inflammatory infiltration, supporting the diagnosis of Tolosa–Hunt syndrome. The diagnosis was based on characteristic imaging findings, exclusion of alternative etiologies, and a prompt response to corticosteroid therapy, consistent with Tolosa–Hunt syndrome.

#### 3.1.3. Case 3: Lymphoma Masquerading as Benign Tumor

**Figure 2:**
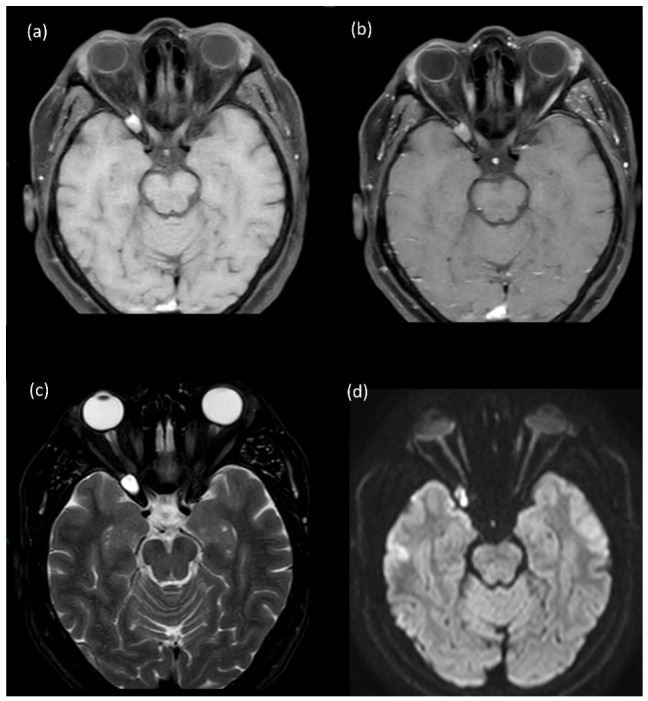
Case 3. Axial MRI demonstrating a right orbital apex lesion measuring 11.5 × 7.1 × 11.3 mm. (**a**) Post-contrast T1-weighted image showing notable enhancement of the lesion. (**b**) Fat-suppressed post-contrast T1-weighted image confirming lesion enhancement. (**c**) T2-weighted image delineating lesion extent. (**d**) T2-FLAIR image demonstrating a solid lesion with surrounding signal alteration.

A 58-year-old woman presented with right eye pain, progressive ophthalmoplegia, reduced VA to 20/40, ptosis, and diminished color saturation. MRI revealed enhanced lesion at the orbital apex suggestive initially of inflammation or epidermoid cyst (Figure 2a–d). She responded well to steroids, but symptoms recurred after cessation of the treatment. Subsequent positron emission tomography-CT and biopsy identified non-Hodgkin’s lymphoma via supraclavicular lymph node sampling. She was referred for hematological–oncological evaluation and treatment. The diagnosis was confirmed histopathologically.

#### 3.1.4. Case 4: Partial OAS Secondary to Acute-Onset Sinusitis

**Figure 3:**
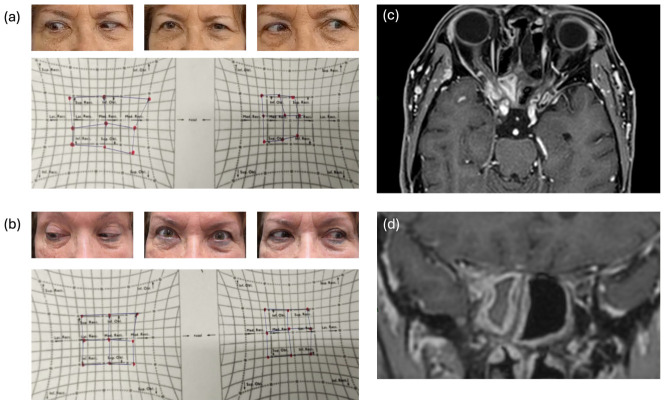
Case 4. (**a**) Gaze positions and Hess screen at presentation demonstrating right eye abduction deficit. (**b**) Post-treatment gaze positions and Hess screen showing near-complete recovery with minimal residual abduction limitation. (**c**,**d**) Contrast-enhanced, fat-suppressed T1-weighted MRI in axial and coronal planes demonstrating right sphenoidal sinusitis with extension to the orbital apex and cavernous sinus.

A 73-year-old female with controlled asthma presented acutely with right optic neuropathy, CN VI palsy, and mild orbital pain after a recent herpes zoster vaccination (HZV) and cataract surgery (Figure 3a,b). Imaging demonstrated orbital apex inflammation and sphenoidal sinusitis (Figure 3c,d). Urgent sphenoidotomy was performed, and the patient was initially treated for suspected fungal infection; however, cultures later grew only skin flora. The etiology was considered infectious based on imaging findings, clinical presentation, and rapid clinical improvement following surgical intervention, despite the absence of definitive microbiologic confirmation.

Surgery led to rapid improvement in VA to 20/20, resolution of neuropathy, and restoration of near-full ocular movements.

#### 3.1.5. Case 5: Blunt Orbital Trauma

A healthy 14-year-old girl presented with acute left eye vision loss (20/400), complete ophthalmoplegia and ptosis, with fixed dilated pupil after blunt orbital trauma. Imaging indicated orbital apex edema without fractures. The diagnosis was based on a clear history of trauma and supportive imaging findings. Lateral canthotomy was urgently performed for suspected compartment syndrome, followed by high-dose steroids. Vision rapidly improved to 20/40 within three days, with gradual but nearly complete recovery of ophthalmoplegia and ptosis over the following five months.

Examination at our outpatient clinic revealed partial ophthalmoplegia in the left eye with left exotropia measured at −40 PD in primary gaze and −45 PD on left gaze with 7 PD right hypertropia.

#### 3.1.6. Case 6: Post-Surgical OAS of Undetermined Etiology

A 78-year-old male patient developed right eye ophthalmoplegia with visual decline (HM) and ptosis approximately one week after transsphenoidal resection of a pituitary macroadenoma. The presentation included optic neuropathy with positive RAPD, CN III, IV, and VI palsies. No abnormalities were noted on anterior and posterior segment eye exam. Imaging (CT, MRI) demonstrated enhancement and inflammatory changes at the right orbital apex, without evidence of mucormycosis. C-reactive protein was elevated but later decreased with oral prednisone. Extensive differential diagnosis considered infection, inflammation, hemorrhage, ischemia, and osteomyelitis. The patient was treated empirically with high-dose corticosteroids, antibiotics, antivirals, and antifungals.

Follow-up showed gradual improvement: VA improved to 20/60 with recovery of eye movements, though a persistent color vision deficit remained. Facial nerve palsy developed on the contralateral (left) side, with significant corneal dryness requiring ocular surface management. Biopsy was deferred due to high procedural risk and evidence of clinical improvement. Final assessment favored post-surgical orbital apex syndrome of undetermined etiology. Infectious and inflammatory causes were considered in the differential diagnosis, but no specific cause could be confirmed.

## 4. Discussion

The present study describes six cases of reversible OAS across diverse etiologies, focusing specifically on a subset of patients with favorable outcomes that is underrepresented in the literature. Median time to clinical improvement from presentation was 2.37 months (range 0.25–5 months). OAS was caused by diverse etiologies, including two inflammatory cases and one case each of lymphoma, infectious sinusitis, blunt trauma, and post-surgical OAS of undetermined etiology, each with typical and atypical features. Remarkably, VA improved in all of them by the end of follow-up.

The literature on OAS suggests mainly unfavorable outcomes, with only one-third of patients experiencing improved VA [4,5]. However, clinical outcomes may vary substantially according to etiology, and favorable outcomes may be more common in selected subgroups. Tumor-related OAS cases seem to carry a poor visual prognosis in about half of the cases [5]. In contrast, in our cohort, the third patient, ultimately diagnosed with non-Hodgkin lymphoma, unusually regained CN II and III function. To note, amblyopia and esotropia precluded assessment of CN VI recovery. Inflammatory cases tend to have a reversible outcome, provided corticosteroid therapy is initiated promptly. Our patient, in whom infection and neoplasm were excluded after extensive workup, showed marked improvement with steroid treatment, as is typical in such cases [4,5]. Compared to prior series reporting predominantly poor outcomes, our findings highlight variability in clinical course and suggest that favorable outcomes may be more common in selected subgroups.

Cranial nerve deficits are common and occur in 62% to 80% of OAS patients [4,5]. In the present series, symptom onset was abrupt in most cases, with multiple simultaneous cranial neuropathies. Two cases differed, with CN VI palsy preceding optic neuropathy. Patient 1 was diagnosed with inflammation-induced OAS, while patient 4 had infectious sinusitis slowly progressing to involve the apex. Notably, these patients (1 and 4) demonstrated the fastest recovery times (1 month and 1 week, respectively) with concurrent resolution of the ophthalmoplegia and optic neuropathy. Prompt surgical intervention led to recovery of full visual function and ocular motility. This contrasts with previous reports of a poor prognosis in similar cases [5,8]. In one report, 1/6 patients experienced a decrease in vision to no light perception despite appropriate treatment [8]. In a cohort of patients with OAS, three cases were attributed to sinusitis [5]. VA worsened in two of them, from 20/200 to light perception and from 20/100 to 20/200, and improved slightly in the third, from 20/63 to 20/50. Notably, the third patient did not present with ophthalmoplegia.

Patient 5, with traumatic OAS, showed the slowest and most indolent recovery (5 months). The CN II recovered first, within 3 days of presentation and treatment, followed by CN IV and CN VI at 3 weeks, until complete recovery. This pattern is consistent with previous case series [9,10]. However, most cases in the literature carry an unfavorable prognosis. A meta-analysis involving 117 patients, the majority of whom were treated surgically and/or medically, reported improvement in VA in half the patients and in ophthalmoplegia, in 85% [11]. Most reported cases necessitated urgent surgical intervention [9,11]. Patients treated with surgical decompression or steroids were more likely to experience an improvement in vision, although ophthalmoplegia persisted. Complete recovery was reported in a case report following a mega-dose corticosteroid regimen, without any adverse events [9]. Nerve growth factor has been suggested as a potential treatment, but the evidence is inconclusive [11]. Treatment in our traumatic case consisted solely of high-dose steroids, avoiding invasive surgical decompression.

The narrowness of the superior orbital fissure (less than 1.6 mm) may pose a risk of reduced compressive tolerance [12]. In patient 5, the width of the left superior orbital fissure measured 2.01 mm, exceeding the susceptible threshold and potentially providing a protective effect.

Relapse was observed in two cases. In case 3, relapse followed premature cessation of steroid therapy. In case 1, the recurrent and familial pattern was notable, as the patient and his mother each experienced self-limited episodes of ophthalmoplegia. However, because the maternal event was anecdotal and no genetic or pathologic confirmation was available, this observation should be interpreted cautiously. At most, it raises the possibility of a shared predisposition or trigger, but no conclusions can be drawn regarding hereditary or environmental mechanisms. The most recent episode occurred during the patient’s pre-wedding period, raising the possibility of a shared environmental or stress trigger, although an inflammatory or autoimmune predisposition cannot be excluded. Recurrent cranial nerve palsies are rare and poorly understood. Prior familial cases suggest possible autosomal dominant inheritance with similar onset and gradual improvement [13], although familial occurrence may also be coincidental or related to botulism [14], metabolic disease, or a rare genetic condition [3]. Potential inflammatory associations include eosinophilic granulomatosis with polyangiitis [15,16], sarcoidosis [17,18], and Rosai–Dorfman syndrome [19,20], but these remain speculative and are unlikely in our cohort given the lack of systemic features. These observations are hypothesis-generating and do not allow for conclusions regarding hereditary or environmental factors.

From a clinical perspective, these findings underscore the importance of early recognition and prompt evaluation of suspected orbital apex syndrome. Although OAS is often associated with poor prognosis, our series highlights that timely, etiology-directed treatment may lead to substantial recovery in selected cases. This supports a proactive diagnostic and therapeutic approach, including urgent imaging, multidisciplinary evaluation, and early initiation of targeted therapy when indicated.

This study has several important limitations. The sample size is small and reflects a selected cohort of patients with favorable outcomes, which limits generalizability. As a result, the findings do not allow for reliable estimation of prognosis or the relative frequency of reversible presentations in orbital apex syndrome. The retrospective design introduces potential biases, including incomplete data capture and variability in diagnostic evaluation and treatment. The absence of a control group precludes comparison with non-reversible cases, limiting the ability to identify factors associated with recovery. In addition, follow-up duration varied between patients, which may affect assessment of long-term outcomes. Importantly, selection bias toward cases demonstrating clinical improvement may overestimate the likelihood of recovery. Accordingly, these findings should be interpreted as descriptive and hypothesis-generating.

## 5. Conclusions

This case series describes a subset of patients with orbital apex syndrome who demonstrated substantial recovery of visual function and ocular motility across diverse etiologies. These findings suggest that favorable outcomes are possible in selected cases, particularly with prompt diagnosis and etiology-directed management. Given the small sample size and selective nature of this cohort, these observations should be interpreted with caution and are primarily descriptive. No conclusions can be drawn regarding overall prognosis or underlying genetic or environmental factors. Further studies in larger, unselected populations are needed to better define prognostic determinants in orbital apex syndrome.

## Figures and Tables

**Table 1:** Demographic and clinical characteristics of six patients with reversible orbital apex syndrome.

Characteristic		Number of Patients	Percentage
Sex	Male	3	50%
	Female	3	50%
Age (years)	Median (range)	65.5 (14–87)	
Laterality	Right eye	3	50%
	Left eye	3	50%
Cranial nerve involved	CN II	6	100%
	CN III	5	83%
	CN IV	2	33%
	CN V	1	17%
	CN VI	6	100%
Symptoms	Visual acuity decline	6	100%
	Pain	5	83%
	Recurrence	2	33%
	Proptosis	3	50%
Diagnosis	Inflammatory	2	33%
	Trauma	1	17%
	Malignancy	1	17%
	Infectious	1	17%
	Post-surgical (undetermined etiology)	1	17%
Treatment	Steroids	5	83%
	Antibiotics/antifungal	4	67%
	Chemotherapy	1	17%
	Surgery	2	33%
Follow-up time (months), median (range)		7 (2–31)	
Time to clinical improvement (months), median (range)		2.37 (0.25–5)	
VA before treatment		20/30–HM	
VA after treatment		20/15–20/60	

CN, cranial nerve. HM, hand motion. VA, visual acuity.

**Table 2:** Clinical characteristics, treatments, and outcomes of six patients with reversible orbital apex syndrome.

No.	Age (yr)	Sex	Eye	Etiology	Clinical Course							Visual Acuity					
					Steroids	Antibiotics/Antifungals	Surgery	CN Involvement (by Order) ^1^	CN Recovery (by Order) ^1^	F-U Time (mo)	Time to Clinical Improvement (mo)	Reduced V1 Sensation	Recurrent Events	Proptosis	Pain	Initial VA	Final VA
1	25	M	OS	Inflammatory (suspected)	+	+		VI → (II, III)	(II, III, VI)	31	1	−	CN VI palsy	+	−	20/30	20/15
2	87	M	OS	Inflammatory (Tolosa–Hunt syndrome)	+	+		(II, III) → VI	III → II → VI *	5	1.75	−	−	+	+	HM	20/40
3	58	F	OD	Lymphoma NHL	+			(II, III, VI)	(III, VI) → II **	9	3	+	+	−	+	20/40	20/25
4	73	F	OD	Infectious sinusitis		+	+	VI → II	(II, VI)	2	0.25	−	−	−	+	20/30	20/20
5	14	F	OS	Blunt trauma	+		+	(II, III, IV, VI)	II → IV, VI → III (Sup. before Inf. division)	13	5	−	−	+	+	20/400	20/40
6	78	M	OD	Post-surgical OAS (undetermined etiology)	+	+		(II, III, IV, VI)	(III, IV, VI) → II	5	5	−	−	−	+	HM	20/60

Abbreviations: CN, cranial nerve; F-U, follow-up; Inf., inferior; NHL, non-Hodgkin lymphoma; OD, oculus dexter (right eye); OS, oculus sinister (left eye); Sup., superior; VA, visual acuity. ^1^ Parentheses denote simultaneous presentation/resolution. * Mild residual deficit at final follow-up. ** Amblyopic eye.

## Data Availability

Data will be made available from the corresponding author upon request.

## Abbreviations

The following abbreviations are used in this manuscript:

CT	Computed tomography
HSV1	Herpes simplex virus 1
HM	Hand motion
HZV	Herpes zoster vaccine
IgE	Immunoglobulin E
MRI	Magnetic resonance imaging
OAS	Orbital apex syndrome
RDS	Rosai–Dorfman syndrome
VA	Visual acuity
